# Familial Hypercholesterolemia: Still an Enigma

**DOI:** 10.1016/j.jaccas.2025.104755

**Published:** 2025-08-27

**Authors:** Sahej Arora, Adnan Kharsa, Gaurav Sharma

**Affiliations:** a Department of Internal Medicine, Rochester General Hospital, Rochester, New York, USA; b Sands Constellation Heart Institute, Rochester General Hospital, Rochester, New York, USA

**Keywords:** atherosclerosis, cardiovascular prevention, familial hypercholesterolemia, statins

## Abstract

**Background:**

Familial hypercholesterolemia (FH) is a genetic condition characterized by high levels of low-density lipoprotein (LDL) and early atherosclerotic cardiovascular disease.

**Case Summary:**

Our patient was a 61-year-old woman who had been referred to a cardiologist for LDL levels >200 mg/dL for more than a decade. She tested positive for a pathogenic *LDLR* mutation and was diagnosed with FH. She then underwent risk stratification with coronary computed tomography angiography and ultrasound of the carotid arteries, both of which showed no atherosclerotic disease. She continues to do well off statins.

**Discussion:**

Coronary artery calcifications can be seen as early as 11 to 23 years of age in patients with FH. Our patient did not have any evidence of atherosclerotic disease, nor did she have a family history of cardiovascular disease, and it was thought that she may have a protective factor affecting LDL metabolism.

**Take-Home Message:**

Patients with heterozygous FH can have absence of atherosclerosis despite lifelong severely elevated LDL levels.

## Graphical Abstract

**Figure:**
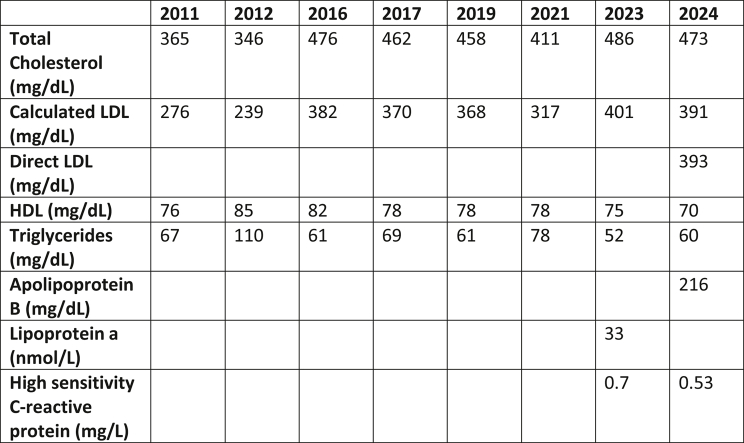

## History of Presentation

**Figure 1:**
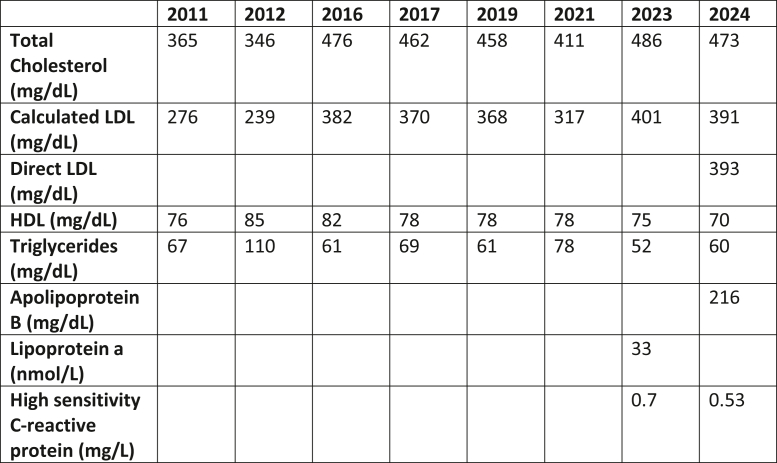
Trends in the Patient's Lipid Levels Across the Years HDL = high-density lipoprotein; LDL = low-density lipoprotein.

A 61-year-old woman was referred to a cardiologist for persistently elevated low-density lipoprotein (LDL) levels. She has had total cholesterol levels of >350 mg/dL and LDL levels of >200 mg/dL for at least the past 12 years (Figure 1), with an LDL of 401 mg/dL at her first presentation to the cardiology office. She had no known personal or family history of cardiovascular disease. Her physical examination revealed corneal arcus but was otherwise unremarkable. She was prescribed a high-intensity statin.

**Take-Home Message**

• Patients with heterozygous FH can have absence of atherosclerosis despite lifelong severely elevated LDL levels.

## Past Medical History

The patient had a past medical history of obstructive sleep apnea.

## Social History

The patient had a prior history of tobacco use (6 pack years of cigarettes that she quit at age 21). She also had a history of alcohol use (2-3 drinks/d for roughly 38 years; she quit completely at age 50). Her diet before presenting to the cardiology office consisted of frequent red meat intake (approximately 4 times/wk), mainly in the form of venison.

## Family History

She had no family history of premature cardiovascular disease. Her mother had a transient ischemic attack at age 82 with right carotid artery stenosis (she is reportedly a “heavy” alcohol consumer, with documented cholesterol levels normal per patient), her maternal grandfather had a stroke at age 95, and her daughter was diagnosed with hypercholesterolemia at age 34; otherwise, her son, 2 siblings, father (all of these family members have reportedly normal documented LDL levels per patient), and other grandparents (documented LDL levels unavailable) have had no known atherosclerotic cardiovascular disease/events. Her grandparents and father are deceased; all of them survived until >70 years of age.

## Investigations

**Figure 2:**
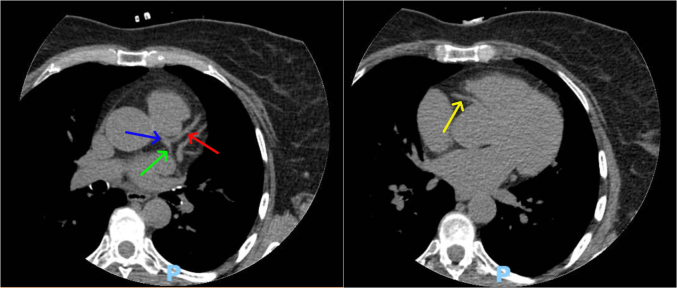
Calcium Score on Computed Tomography Showing Absence of Coronary Calcifications in the LMCA (blue arrow), LAD (red arrow), LCx (green arrow), and RCA (yellow arrow) LAD = left anterior descending artery; LCx = left circumflex artery; LMCA = left main coronary artery; RCA = right coronary artery.

**Figure 3:**
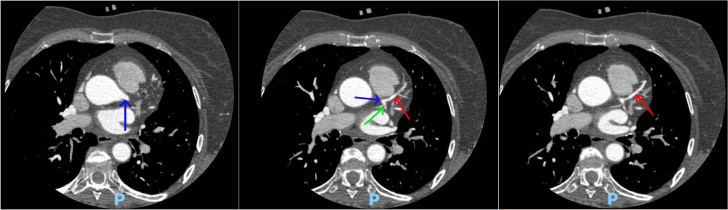
Coronary Computed Tomography Angiography Showing No Soft or Calcified Plaques in the LMCA (blue arrow), LAD (red arrow), or LCx (green arrow) Abbreviations as in Figure 3.

**Figure 4:**
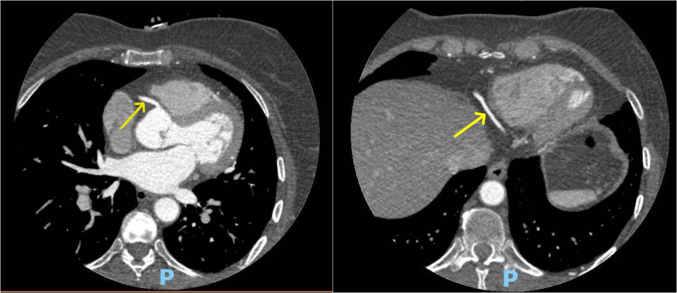
Coronary Computed Tomography Angiography Showing No Soft or Calcified Plaques in the RCA (yellow arrow) RCA = right coronary artery.

**Figure 5:**
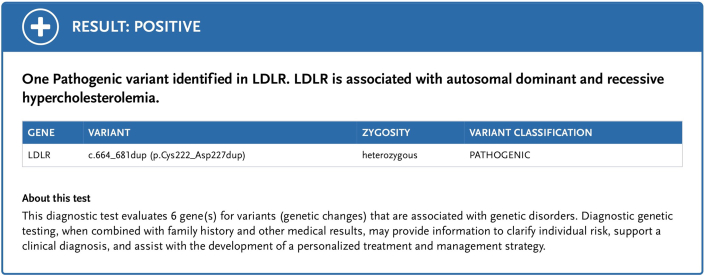
Genetic Testing Result for the *LDLR* Mutation

Testing was pursued for risk stratification to decide further course of action. High-sensitivity C-reactive protein level was 0.53 mg/L (normal: <1.0 mg/L), lipoprotein A level was 33 nmol/L (normal: <75 nmol/L), and apolipoprotein B level was 216 mg/dL (normal: <80 mg/dL). Coronary artery calcium score was zero (Figure 2). We further obtained coronary computed tomography angiography (CCTA) (Figures 3 and 4) and ultrasound of the carotids to evaluate for any evidence of subclinical atherosclerosis; both tests showed no atherosclerotic plaques. Genetic testing revealed a pathogenic heterozygous mutation of the *LDLR* gene (variant c.664_681dup (p.Cys222_Asp227dup)) (Figure 5) consistent with heterozygous FH.

## Management

Based on her LDL levels, our patient was started on high-intensity statin therapy before risk stratification. However, after obtaining her CCTA and carotid ultrasound, she chose to come off statins given a lack of evidence for atherosclerotic cardiovascular disease despite longstanding, markedly elevated LDL levels. We decided to monitor her coronary anatomy every 2 years with CCTA.

## Outcome and Follow-Up

The patient continues to do well 2 years after her diagnosis and without any lipid-lowering therapy.

## Discussion

FH is an autosomal dominant disorder characterized by mutations in ≥1 genes responsible for LDL catabolism.^1^ It is characterized by markedly elevated LDL levels and propensity for early atherosclerotic cardiovascular disease. Heterozygous FH has an estimated prevalence of approximately 1 in 200 people.^2^ Patients with heterozygous FH also have premature atherosclerotic cardiovascular disease and may have up to 20-fold increased risk if left untreated. Coronary artery calcifications, which are uncommonly seen before the fourth decade of life, can be identified at 11 to 23 years of age in these patients.^3^ Diagnosis involves many components, ranging from detailed history to genetic testing, and it employs various scoring systems, of which the most widely used is the Dutch Lipid Clinic Network Criteria. Intense LDL-lowering therapy decreases coronary atherosclerosis^4^ and reduces cardiovascular events,^5^ coronary heart disease–related mortality,^6^ as well as all-cause mortality^7^; hence, all patients are offered aggressive therapy as soon as diagnosis is made.

Our patient had a history of longstanding derangements in her LDL levels and tested positive for a pathogenic heterozygous mutation of the *LDLR* gene. Her LDL level at presentation was 401 mg/dL, with levels consistently >250 mg/dL since 2011. Despite her longstanding and severely elevated LDL level, she did not have any evidence of coronary or carotid atherosclerosis.

Although the absence of coronary artery calcium in middle-aged patients with FH is not uncommon,^8^ it does not rule out noncalcified plaques, which can be assessed by CCTA. Absence of coronary plaque in patients with FH has been previously described in a pooled analysis,^8^ in which the authors found that coronary artery calcium is heterogenous in this population, with nearly half of the patients with FH demonstrating no coronary artery calcium. The absence of coronary calcium among individuals with FH is considered a favorable prognostic factor. One study^9^ found that patients with FH undergoing statin treatment with a coronary artery calcium score of zero did not have any cardiovascular events over a mean follow-up period of 3.7 years, and all cardiovascular events occurred in patients who had coronary artery calcium present.

It is unclear why some patients with FH have absence of coronary atherosclerosis despite having lifelong severely elevated LDL levels. Atherosclerosis is a multifactorial disease, and other concomitant risk factors may be contributing.^10^ Miname et al^9^ found that traditional risk factors were predictors of atherosclerosis disease in patients with FH; these risk factors included male sex, older age, diabetes, hypertension, higher body weight, elevated lipoprotein A levels, and having started statin therapy at an older age. Our patient's risk factors consisted of class I obesity, prior history of tobacco use, alcohol use, and red meat intake, with normal lipoprotein A and high-sensitivity C-reactive protein levels. She had no family history of premature cardiovascular disease, and this may suggest a possible “protective” genetic factor that may affect or alter the LDL particle morphology and function, LDL oxidation process, macrophage function, or inflammation, counteracting the effects of the *LDLR* mutation and preventing atherosclerosis. Vascular age derived from coronary artery calcium score could be used alone or with tools such as the SAFEHEART (Spanish Familial Hypercholesterolaemia cohort study) risk equation to stratify patients as having lower or higher risk.

One follow-up study by Pérez de Isla et al^11^ of patients with FH found a “resilient familial hypercholesterolemia” phenotype of patients aged ≥65 years but without clinical atherosclerotic vascular disease. These patients were associated with a defective LDL-receptor mutation, higher levels of high-density lipoprotein, younger age, lower score on the SAFEHEART risk equation, absence of hypertension, and lower lipoprotein A levels. In a study by Melnes et al^12^ these patients had increased expression of *ABCA1* and *ABCG1* membrane transporters regulating cholesterol efflux and were associated with higher concentrations and larger particle size of high-density lipoproteins. CCTA can be used to evaluate for noncalcified plaques in these patients as well. The current literature lacks long-term studies to determine risk of cardiovascular events in this population, and further studies are needed to evaluate risk stratification strategies and subsequent management.

We decided to monitor our patient off statins with a CCTA every 2 years, consistent with recommendations from American Heart Association/American College of Cardiology 2021 chest pain guidelines on “warranty period” of CCTA. Because our patient had already spent many years with her elevated LDL levels and was angiographically without evidence of coronary artery disease, we were comfortable with this interval. More studies are needed to determine the warranty period for CCTA in patients with FH without coronary disease.

## Conclusions

Patients with gene-positive heterozygous FH can have absence of atherosclerosis despite lifelong severely elevated LDL levels. The exact mechanism is unclear, however concomitant risk factors along with possible “protective” gene mutations may be contributing. Further studies are needed to understand this paradox, as they may offer non–LDL mediated therapies for the prevention and management of atherosclerosis.

## Funding Support and Author Disclosures

The authors have reported that they have no relationships relevant to the contents of this paper to disclose.
